# Microbial enhancement of crop abiotic stress tolerance: mechanisms and applications

**DOI:** 10.3389/fpls.2026.1992134

**Published:** 2026-10-29

**Authors:** Uzma Aslam, Xueqi Liang, Yongliang Yan, Mengyang Li, Mengke Du, Tingyong Mao, Mohammad Nauman Khan, Lili Yang, Yunlong Zhai

**Affiliations:** 1 College of Agriculture, Tarim University, Alar, China; 2 Key Laboratory of Genetic Improvement and Efficient Production for Specialty Crops in Arid Southern Xinjiang of Xinjiang Corps, Alar, China; 3 Academy of Agricultural Sciences of Xinjiang Uyghur Autonomous Region, Urumqi, China; 4 Laboratory of Plant Molecular Genetics & Crop Gene Editing, School of Life Sciences, Linyi University, Linyi, China; 5 College of Biological Science and Technology, Yili Normal University, Yining, China

**Keywords:** abiotic stress, climate change, heavy metals, microorganisms, plant-microbe interaction

## Abstract

Crops aimed at nourishing an exponentially increasing population are frequently vulnerable to a diversity of adverse ecological conditions. Due to climate change, extreme events including flooding, droughts, heat, and frost are expected to further upsurge in the future. To cope with such effects, a wide range of mitigation and adaptation approaches are needed. Even though plants have been engineered to endure stressful conditions, and some have also developed ways of regulating their metabolism, there is still a scarcity of food supply. To mitigate abiotic stresses, an alternate strategy is to investigate the possibility of utilizing rhizosphere microbes, as it is a cost-effective biological approach for abiotic stress management, which can be employed on a short-term basis. In this review, the various mechanisms, including promoting plant growth, up-regulating the expression of antioxidant and dehydration response genes, reducing ethylene levels, and stimulating the production of soluble phosphates and plant hormones by which microorganisms regulate plants’ reactions to different abiotic stresses, such as high temperature, drought, low temperature, metal toxicity damage, and salinity, are discussed. With this review, we aim to encourage the strategy of using microbial technology to feed the world’s population under the existing resource conditions and propose that the improvement of crops’ abiotic stress tolerance by microbial technology is the key to making crops more adaptable to future climatic conditions and proposing it as promising and feasible future technology.

## Graphical Abstract

**Figure:**
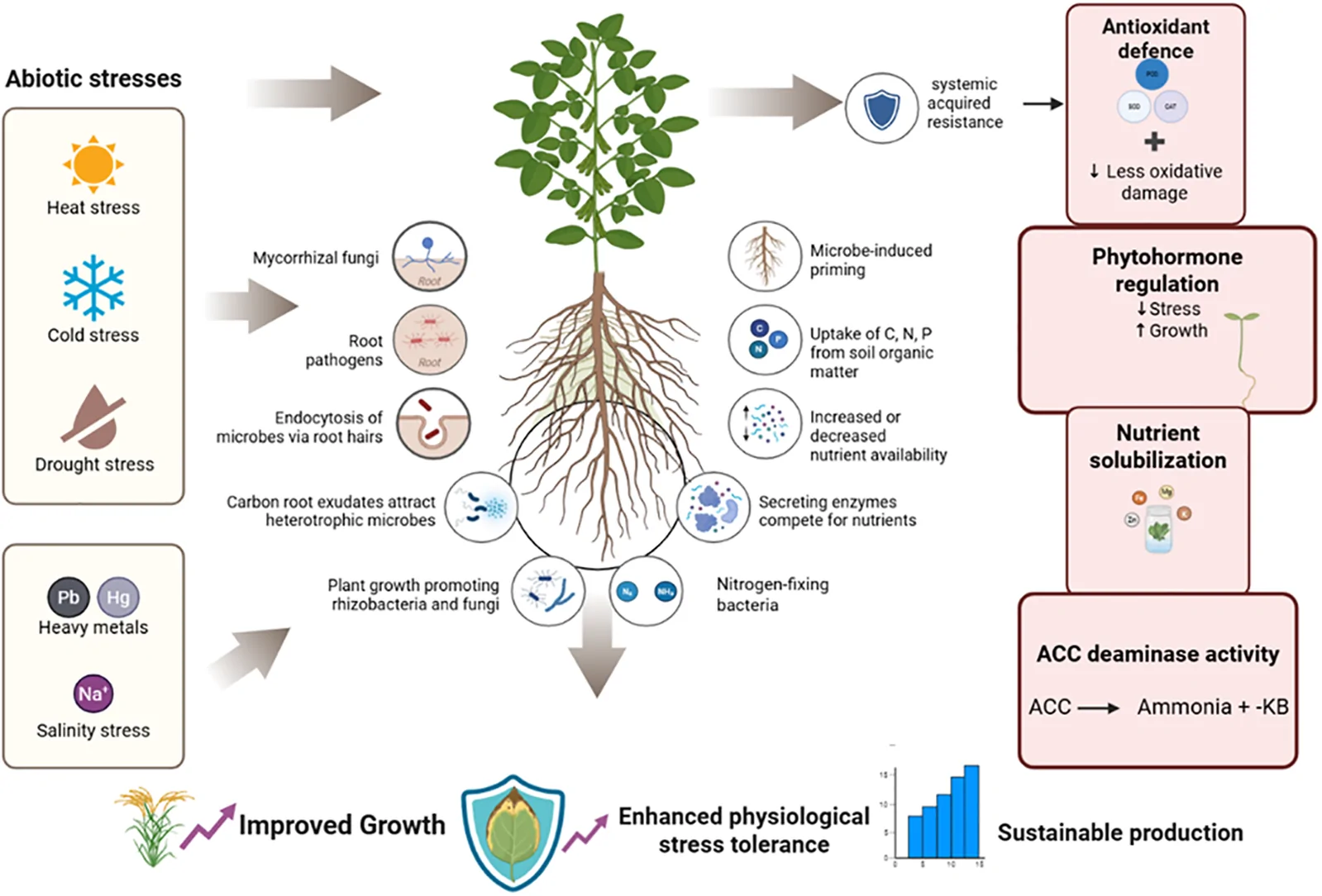

## 1 Introduction

Urgent attention is needed to handle the effects of climate change on agricultural crops, usually caused by erratic rainfall patterns, a rise in temperature, and soil salinity. As these damages have adverse effects on crops, a famine becomes a major challenge for humans, which results in food security threats. Abiotic stresses (heat, cold, heavy metal, drought and salinity stress) harshly affect crop growth, hindering their productivity. Around 90% of the world’s farmland is affected by one or more dangerous abiotic stresses that lead to yield loss of up to 70% (Waqas et al., 2019; Liu et al., 2020; Gui et al., 2021). These damages are abundant, whereby temperature stress counts 20%, 7% from chilling stress, drought stress counts 9%, and other forms of stresses amounts 4% (Minhas et al., 2017; Luqman et al., 2023; Sajid et al., 2023a). Economically, these stresses cause a loss of more than a hundred million dollars, and every year, a loss of twenty-seven billion dollars is caused by soil salinity. Surveys predict that climate instability might lead to a 40-60%reduction in cereals and oilseeds crop yields by 2090 (Luqman et al., 2023; Sajid et al., 2023a, b). Due to global warming, extreme weather events, floods, and drought conditions are happening more often, which badly impact farmland productivity. Verma et al. (2022) estimated that abiotic stresses may reduce annual food crop production by 51–82%, highlighting the pressing need for effective mitigation strategies to ensure yield stability, food security, and long-term agricultural sustainability. Meanwhile, the global population continues to rise, intensifying the demand for food, while current production levels remain insufficient to meet this demand. In arid and semi-arid regions, efforts to enhance crop production have led to increased reliance on surface and groundwater resources for irrigation, consequently exacerbating soil salinization. Therefore, we need to find ways to help crops resist abiotic stress, which should be economical, environmentally friendly, and effective.

Plants have developed mechanisms to adapt to stressful environments with well-known responses including osmolyte synthesis, modification of water transport, and elimination of reactive oxygen species (Fadiji et al., 2022). Techniques in plant biotechnology have been applied to create crop varieties having disease resistance, stress tolerance, and most importantly, enhanced nutritional quality (Zhao et al., 2020). While these approaches can be used to develop genetically modified crops or plants that tolerate stressful environmental conditions, their high cost and restricted access are likely confined to developed countries whereby still a limiting factor for underdeveloped countries. In parallel, the associations between soil biota and plants play a fundamental role in sustaining terrestrial ecosystems and shaping how they respond to shifts in climate (Cavicchioli et al., 2019). Certain microorganisms play a role in agriculture and are currently utilized in cultivating sustainable food crops (Ferreira C et al., 2019; Kassam et al., 2019). Research indicates that these advantageous microbes alleviate stress through several mechanisms, such as fostering growth by enhancing phytohormone synthesis, siderophore production, and phosphate solubilization; reducing ethylene concentrations and improving the genes activity involved in dehydration response and antioxidant defense (Ojuederie et al., 2019).

Recent investigations focused on harnessing rhizosphere microbial communities to improve the sustainability of food production (Bender et al., 2016; Thirkell et al., 2017). It is stated that dormancy and elicited-seedling growth have been induced by root-colonizing bacteria that produce phytohormones, which can alleviate soil salinity Kushwaha et al. (2020). Kumar et al. (2021) used PGPR *Bacillus* and *Pseudomonas* sp. in stressed plants to enhance growth rate by the production of phosphates, indole acetic acid (IAA), and siderophores. Kaushal (2019) demonstrated that via increasing the expression of the early responsive to dehydration 15 (*ERD15*) gene, *Paenibacillus polymyxa* rhizobacterium increases drought stress resilience. Zhang et al. (2020) found that the arbuscular mycorrhizal (AM) fungus enhanced growth, biomass yield, and osmotic regulation (through elevated absorption of potassium, calcium, and magnesium ions), affecting the citrus tangerine species. Certain PGPR strains have additionally demonstrated the ability to enhance stress resistance by generating antioxidants that break down reactive oxygen species (ROS) (Hasanuzzaman et al., 2021). AM fungal hyphae being capable of accessing soil pores to root hairs, absorbing water and nutrients unavailable to non-AM plants (Li et al., 2019). Thus, they enhance plant growth by modifying plant–water relations, leading to improved water use efficiency and, consequently, higher crop yields (Adedeji and Babalola, 2020). Beneficial mycorrhizal strains are occasionally applied in combination with PGPR to induce the effectiveness of plant growth promotion, making the use of mycorrhizal symbiosis utilization more attractive for agricultural productivity that is sustainable (Kaur and Suseela, 2020).

In recent years, swift technological and theoretical advances in soil microbiology have elucidated the impacts of the soil microbiome on different plant stress resistance. Several studies indicate that microorganisms alleviate stress primarily by stimulating core metabolic processes like nutrient absorption, plant growth, antioxidant enzyme activity and photosynthesis. There is also proof suggesting a role for secondary metabolites (Mohammadi et al., 2021). Plants under stress showed certain secondary metabolites, including phytoalexins, phenylpropanoids, flavonoids, and carotenoids, that were inoculated with microbes (Chukwuneme et al., 2021a, b). These secondary compounds operate as antioxidants that neutralize ROS that assist plants in withstanding pressure (Kour et al., 2020). Bacteria and fungi both are frequently associated with the induction of secondary metabolites in plants under stress. Hence, the soil biota is highly heterogeneous, making it difficult to fully characterize the complex and dynamic interactions that occur within it. Various factors affect the colonization of plant roots, such as soil type, temperature, the variety of plants developing in a specific soil and the proportion of different microorganisms in the rhizosphere. Even though colonization patterns of plants by microorganisms, here, positive effects may be obtained considerably as shown in (**Figure 1**).

**Figure 1:**
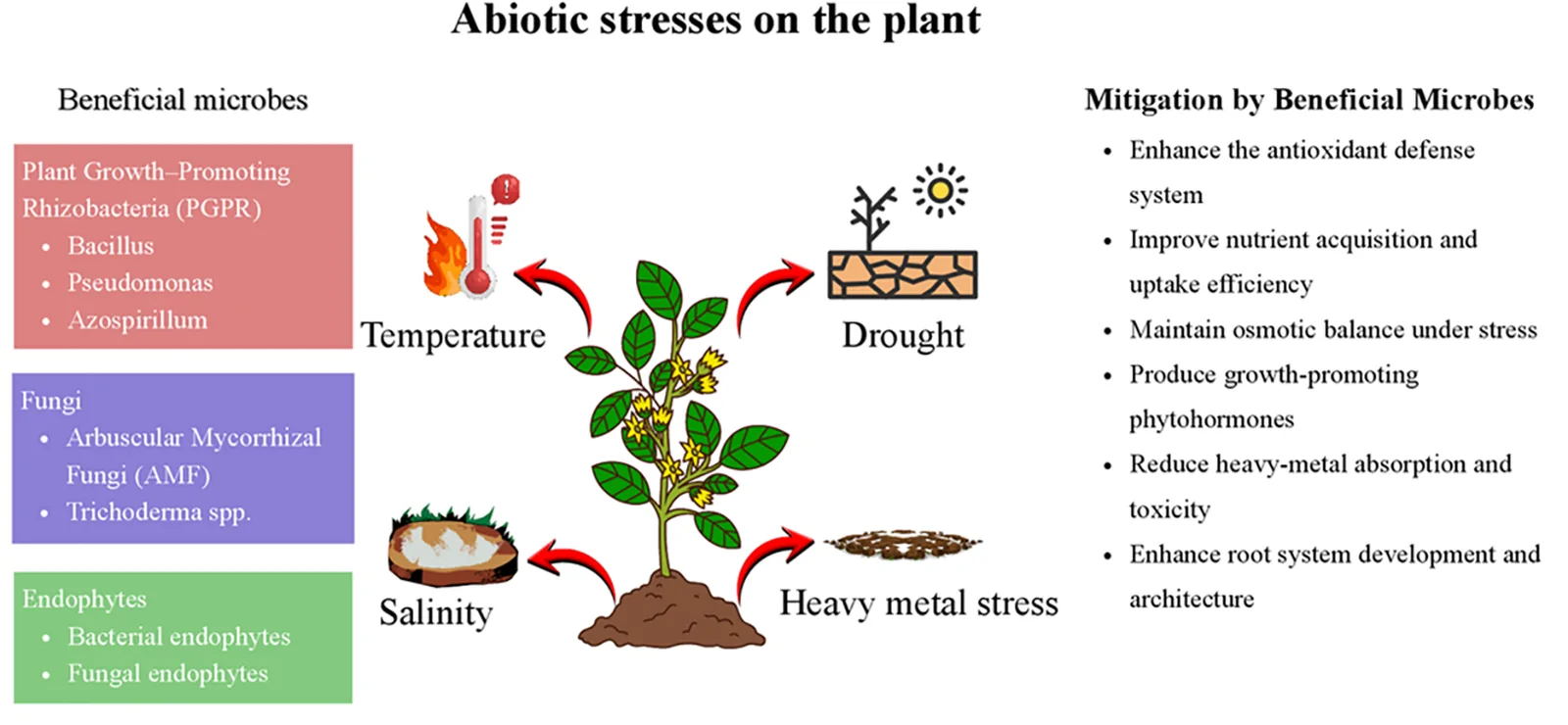
Schematic illustration of major abiotic stresses in plants and the mitigation strategies mediated by beneficial microorganisms such as PGPR, mycorrhizal fungi, and endophytes that enhance antioxidant defense, nutrient uptake, osmotic balance, and root development.

To date, our understanding of the vital processes underlying microbiome-induced plant adaptations under various stresses remains partial because of the complications inherent in plant cellular systems and in the rhizosphere. Hence, this article highlights the evidence on the advantageous roles of microorganisms and identifies microbial strains that are effective in mitigating the impacts of abiotic stresses under extreme environmental conditions.

## 2 The effects of abiotic stresses on plants and the mechanisms of plant responses to stresses

Understanding how microbial inoculation alters plant metabolism under stress requires approaches that capture molecular-level changes. Integrative metabolomic and proteomic analyses have proven valuable for deciphering plant responses to nutrient stress (Moshood et al., 2025) as indicated in **Figure 2**.

**Figure 2:**
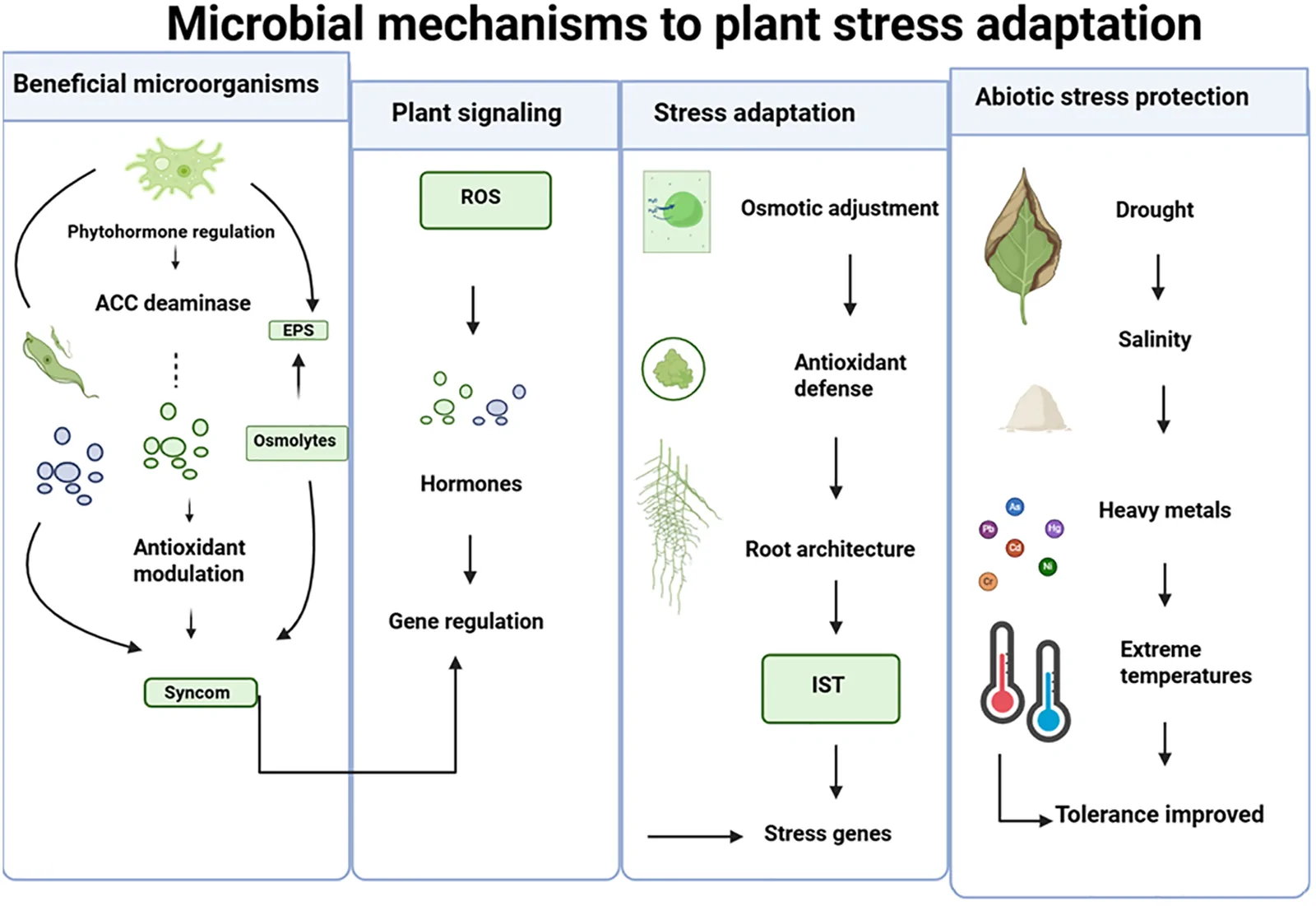
Microbial mechanisms contributing to plant adaptation to abiotic stresses. Beneficial microbes modulate phytohormones, ACC deminease, osmolytes, antioxidants and exopolysaccharides, thereby regulating ROS and stress related signaling, ultimately improving tolerance to drought, salinity, heavy metals and extreme temperatures.

### 2.1 Drought stress

Climate anomalies and the disruption of environmental equilibrium have made water scarcity a critical global concern. Drought is a major impediment to agricultural development, its frequency and intensity are expected to increase under climate change, and it severely reduces crop yield (Khan et al., 2013). By inhibiting stomatal movement, respiration, and photosynthesis, drought disturbs physiological metabolism and plant growth. Plants respond by activating hormone synthesis, drought-responsive gene expression, morphological and structural adjustments, and osmotic regulators (Sato et al., 2024). Drought perception generates signaling molecules, including cyclic ADP-ribose (cADPR), abscisic acid (ABA), Ca²^+^, nitric oxide (NO), and inositol 1,4,5-trisphosphate (IP3), which relay the signal through transduction cascades to downstream genes (Yang X. et al., 2021). Functional gene products such as glycine betaine (GB), proline, soluble sugars (SS), aquaporins (AQPs), and late embryogenesis abundant (LEA) proteins directly influence plant metabolism, whereas regulatory products, including *WRKY*, *MYB*, *NAC*, *AP2/ERF*, and *HD-Zip/bZIP* transcription factors, calcium-dependent protein kinases (CDPKs), and mitogen-activated protein kinases (MAPKs), control signal transduction and downstream gene expression, enabling plants to endure dry conditions (Yang X. et al., 2021; Wu et al., 2022).

Drought-tolerant microbes can sustain plant growth at low water potential. They have adapted to water scarcity by entering a quiescent state, developing thick cell walls, producing exopolysaccharides (EPS), and accumulating osmolytes (Kumar and Verma, 2018). Colonizing the rhizosphere, they support plants through direct and indirect routes irrespective of soil water content, mainly by (1) synthesizing phytohormones such as ABA, indole-3-acetic acid (IAA), and cytokinins, (2) ACC deaminase activity, (3) bacterial EPS, and (4) induced systemic tolerance (Farooq et al., 2009; Porcel et al., 2014a). Among these hormones, ABA is the key regulator under drought, whereas IAA, the most active auxin, governs adventitious and lateral root differentiation, cell division, shoot growth, and vascular development (Porcel et al., 2014a; Goswami et al., 2015).

In bacteria, osmotic and envelope stress are sensed through conserved two-component systems such as EnvZ–OmpR and Rcs, which induce small RNAs (e.g., OmrA, OmrB, MicF in *Escherichia coli* and *Salmonella enterica*) that remodel outer-membrane composition and permeability under water-limiting or osmotically variable conditions (Yi et al., 2026). Such sRNA-mediated stress circuitry, though characterized mainly in enteric and soil-associated genera such as *Pseudomonas* and *Rhodobacter*, offers a mechanistic framework for understanding how rhizosphere bacteria used in microbial inoculants may maintain viability and functionality under drought-induced osmotic stress.

Inoculation with PGPR increases ABA and modulates plant physiology by regulating transcription of genes linked to root hydraulic conductivity and drought response (Jiang et al., 2013) (**Figure 3**). Complementarily, ACC deaminase restricts stress-induced ethylene and sustains root growth under water deficit, while microbial metabolites promote osmo-protectant accumulation and ROS regulation, preserving membrane integrity and limiting oxidative damage during dehydration (Vurukonda et al., 2016; Wang et al., 2024). Microbial control of root architecture and nutrient availability further improves soil exploration and resource acquisition when water is limited.

**Figure 3:**
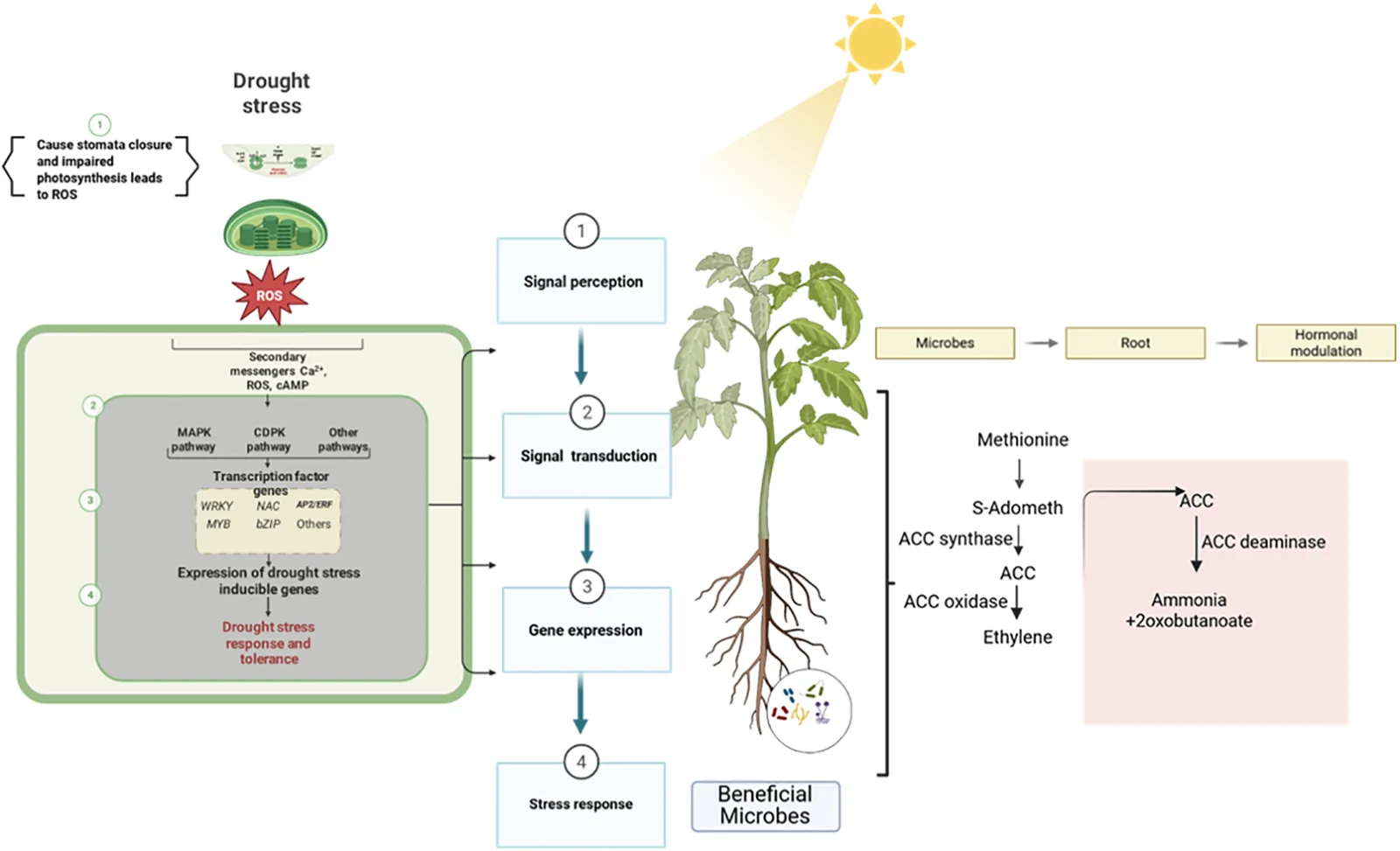
Microbial regulation of drought stress in plants by reducing oxidative damage and modifying ethylene production.

These responses have been conceptualized as rhizobacterial-induced drought endurance and resilience (RIDER), involving coordinated biochemical and physiological changes (Kaushal and Wani, 2016). However, the physiological signatures reported differ between systems. In soybean, *Pseudomonas putida* H-2–3 increased jasmonic acid while lowering salicylic acid, ABA, flavonoids, superoxide dismutase, and radical-scavenging activity (Kang et al., 2014), suggesting that stress was relieved through hormonal rebalancing with a reduced oxidative load. By contrast, in maize, three drought-tolerant strains, *Proteus penneri* (Pp1), *Pseudomonas aeruginosa* (Pa2), and *Alcaligenes faecalis* (AF3), increased protein, sugar, and relative water content together with proline (Naseem and Bano, 2014), pointing to an EPS- and osmolyte-driven route. Similar growth benefits can therefore arise through different physiological routes depending on host and strain, which complicates generalization from individual traits. Effective management of drought stress requires accurate monitoring of soil water status. Advances in dielectric-based soil moisture sensing provide practical tools for quantifying water availability in the root zone (Abdulraheem et al., 2024). Combining such monitoring with microbial inoculation strategies could help optimize the timing and placement of beneficial microorganisms under field conditions.

Recent perspectives describe drought mitigation as a molecular dialogue between plants and their microbiota, in which microbial signaling molecules and metabolites activate drought-responsive and osmo-protective pathways (Gahlot, 2026). Inoculation modulates genes involved in stress perception, hormone signaling, osmotic adjustment, antioxidant defense, and cellular protection (Gahlot, 2026; Wang et al., 2024), and genomic, transcriptomic, proteomic, and metabolomic approaches are increasingly used to identify the underlying microbial signals and plant pathways (Gahlot, 2026). The nutritional dimension is illustrated by rhizobial symbiosis in legumes, where efficient nodulation and biological nitrogen fixation sustain plant nutrition and soil fertility under water limitation and thus support growth and productivity during drought (Gahlot, 2026). The need for adaptive solutions to climate change has driven a growing number of studies, many of which apply several strains together on the premise that a consortium can provide synergistic advantages over single strains and approximate the original microbiome (Shaffique et al., 2022). This marks a shift from individual traits toward functional communities, and tailored consortia and precision microbiome engineering have been proposed to combine complementary nutrient-acquisition, root-development, osmoprotection, and signaling functions. Nevertheless, efficacy varies with plant genotype, soil properties, microbial competition, and environmental conditions, so integrating functional traits with multi-omics data and field-scale validation is required to make microbiome-based drought strategies reliable.

### 2.2 Salinity stress tolerance

According to the Food and Agriculture Organization (FAO), over 20% of land areas are affected by salinity (Wagner et al., 2014), and approximately 20% of irrigated farmland worldwide is salinized (Morton et al., 2019). Crop loss from soil salinity is an increasingly pressing problem (Yang and Guo, 2018), driven by rising saline groundwater levels, intensified evaporation under drought, excessive irrigation, climate change, and inputs such as manure, sewage sludge, and municipal garden waste. Sustainable use of saline land is therefore crucial for global food security. Salinity constrains both plant growth and microbial activity mainly through osmotic stress and ion toxicity: Na accumulation triggers chlorosis and disrupts ion balance, lowering yield, and fungi are generally more sensitive to salt than most bacteria. When soil water potential becomes unfavorable, plants and microorganisms alike struggle to extract water, and plants lose turgor, close stomata, and suffer reduced photosynthetic performance (Shaffique et al., 2022).

Plants rapidly detect excess Na^+^ and initiate sodium-stress responses (Choi et al., 2014; Bose et al., 2015). Within two hours of salt exposure, the conserved salt overly sensitive (SOS) pathway is activated, in which SOS2 kinase and the SOS1 Na^+^ antiporter promote Na^+^ efflux (Farooq et al., 2009). Salt also raises ABA concentrations (Lin et al., 2009) and activates sucrose non-fermenting 1-related protein kinase 2 (SnRK2) (Geilfus et al., 2015), and SnRK2 activity increases further in the protein phosphatase 2C double mutant *abi1–2 pp2ca-1* (Krzywinska et al., 2016). Because SnRK2 can be activated by salt or osmotic stress independently of ABA (Zhang et al., 2016), osmotic signaling appears to proceed through both ABA-dependent and -independent pathways without requiring a Na^+^ sensor (Sewelam et al., 2014). In *Arabidopsis*, osmotic stress induces 1,118 genes and salt stress 932, of which 435 are shared, while 364 and 367 genes are repressed, respectively, with 154 overlapping (Sewelam et al., 2014), implying that plants sense salinity through both osmotic and ionic sensors. *HKT1* is a major determinant of tolerance (Platten et al., 2006): by limiting Na^+^ loading into the shoot, it shields leaves from Na^+^ toxicity (Ren et al., 2005; Horie et al., 2009). It is highly expressed in leaf vascular tissues and root cells, and *hkt1* loss-of-function mutants are salt-hypersensitive, with high shoot and reduced root Na^+^, consistent with a role in regulating Na^+^ transport in the xylem sap from root to shoot (Davenport et al., 2007). Tissue-specific enhancement of *HKT1*, for example in the vascular bundle or pericycle, can improve whole-plant salt tolerance (Møller et al., 2009), and *hkt1* mutations suppress the salt hypersensitivity of *sos2* and *sos3* mutants, indicating functional interaction between *HKT1* and the SOS pathway in Na^+^/K^+^ homeostasis (Rus et al., 2001, 2004). Genome-wide association studies (GWASs) and quantitative trait locus (QTL) mapping of natural variation further suggest that *HKT* loci have been selected for salinity resistance during evolution and breeding (Asins et al., 2013; Ariyarathna et al., 2016). These primary ionic and osmotic effects are followed by secondary oxidative stress, in which excess ROS cause protein degradation, DNA mutations, and membrane peroxidation.

Inoculation with PGPR and endophytic microbes alleviates the adverse effects of salinity across a wide diversity of salt-tolerant strains, through interconnected nutritional, hormonal, osmotic, antioxidant, and molecular mechanisms (Vaishnav et al., 2019; Kumar et al., 2020; Vaishnav et al., 2016). Direct mechanisms improve plant nutrition and stress physiology, whereas indirect mechanisms modify the rhizosphere, suppress pathogens, and activate plant defense responses (Vaishnav et al., 2016). Nitrogen fixation, phosphate solubilization, potassium mobilization, and siderophore production improve nutrient availability and help maintain ionic homeostasis (Vaishnav et al., 2019; Kumar et al., 2020; Gopalakrishnan et al., 2015; Ferreira, M et al., 2019), while microbial IAA, cytokinins, and gibberellins increase root number, length, and surface area and hence nutrient uptake under stress (Kumar et al., 2020). A key trait is ACC deaminase, which cleaves the ethylene precursor ACC into ammonia and α-ketobutyrate, restricting stress-induced ethylene and sustaining root and shoot growth (Kumar et al., 2020). Microbial metabolites act in parallel: EPS bind Na^+^ and other ions, improve soil aggregation and water retention, and stabilize root colonization; biofilm formation under saline conditions has been reported to alleviate salt injury effectively (Kasim et al., 2016); and volatile organic compounds (VOCs) act as signals that activate plant stress responses and have been linked to modulation of ion transport through SOS- and HKT-related mechanisms (Kumar et al., 2020; Sunita et al., 2020).

Crop-level studies confirm these mechanisms but differ in the physiological route emphasized. *Azospirillum* improved germination and vegetative growth of lettuce under saline conditions (Barassi et al., 2006), *Pseudomonas stutzeri* mitigated salinity effects in both salt-susceptible and salt-tolerant chili pepper (Bacilio et al., 2016), and biofilm formation contributed to tolerance in barley (Chandrasekaran et al., 2016). In rice and maize, *Bacillus pumilus* and *P. pseudoalcaligenes* enhanced salt tolerance with higher glycine betaine concentrations (Bano and Fatima, 2009; Jha et al., 2011), consistent with the broader enhancement of compatible solutes such as proline, glycine betaine, and soluble sugars in inoculated plants (Kumar et al., 2020; Sunita et al., 2020). Antioxidant responses, however, are less consistent. *Acinetobacter* and *Pseudomonas* spp. decreased superoxide dismutase (SOD) activity and lipid peroxidation in the salt-sensitive rice cultivar GJ-17, thereby alleviating oxidative damage (Chang et al., 2014), whereas other work reports that beneficial microbes stimulate SOD, catalase (CAT), and glutathione reductase (GR) to limit ROS accumulation (Vaishnav et al., 2019, 2016). These opposing trends probably reflect differences in cultivar, stress severity, and sampling time, since lower enzyme activity may indicate a reduced oxidative load whereas higher activity indicates enhanced scavenging capacity, and they caution against using a single antioxidant marker as evidence of mechanism. In barley and oats, ACC deaminase and IAA production during salt stress contributed to enhanced growth (Jha and Subramanian, 2014).

At the molecular level, microbial interactions activate defense-associated enzymes such as phenylalanine ammonia-lyase (PAL) and polyphenol oxidase (PPO) and induce stress-responsive genes including *DREB2b*, *RD29A*, *RD29B*, and *RAB18* (Kumar et al., 2020). Rice inoculated with the salt-tolerant *Bacillus amyloliquefaciens* NBRISN13 (SN13) showed improved tolerance and growth through up- and down-regulation of 14 genes (Nautiyal et al., 2013), and endophytic PGPR-derived hormones combined with exogenous jasmonic acid (JA) alleviated salt stress in *Solanum pimpinellifolium* (Khan et al., 2017). Tricarboxylic acid (TCA) cycle activity has been identified as a further component of salt resistance in tomato (De La Torre-González et al., 2017). Together, these findings indicate that microbial salinity tolerance involves coordinated regulation of hormonal balance, osmotic adjustment, ROS homeostasis, ion transport, and gene expression rather than a single protective mechanism.

Arbuscular mycorrhizal fungi (AMF) provide a complementary, fungal route. Colonization improves growth under salt stress in maize, clover, tomato, sweet basil, cucumber, and lettuce (Porras-Soriano et al., 2009; Elhindi et al., 2017), and in woody fruit crops such as olive, apple, and citrus (Navarro et al., 2014; Yang et al., 2014). Proposed mechanisms include improved host nutrition, a higher K^+^/Na^+^ ratio, osmo-regulator production, greater photosynthetic capacity and water-use efficiency, and facilitated water uptake; AMF also stimulate antioxidant enzymes and influence genes for proline synthesis, AQPs, and LEA proteins (Porcel et al., 2012). Unlike bacterial inoculants, which act mainly through hormones, ACC deaminase, and EPS, AMF act chiefly through nutrition and ion balance, which may explain why co-inoculation can be synergistic: *Rhizophagus intraradices* combined with *Massilia* sp. RK4 improved nutrient accumulation and AMF root colonization in maize under salinity (Chandrasekaran et al., 2016).

Beyond direct inoculation, soil amendments can restructure resident bacterial communities and thereby indirectly deliver comparable functional benefits for salinity tolerance. In saline-alkali soils of the Yellow River Delta, combined straw and desulfurization gypsum application reduced soil pH, sodium adsorption ratio, and dissolved organic carbon, while enhancing sequestered organic carbon, enzyme activity, and aggregate stability. These physicochemical improvements coincided with marked shifts in bacterial community composition and diversity across aggregate fractions (Zhao et al., 2025). *Chloroflexi*, *Actinobacteria*, and *Acidobacteria* were enriched, whereas salt-sensitive *Proteobacteria* and *Bacteroidete*s declined, with microbial biomass carbon and β-glucosidase activity closely tracking these community changes. Collectively, these findings indicate that management practices that alter the soil physicochemical environment, without deliberate microbial introduction, can selectively favor beneficial taxa and enzymatic functions associated with salinity stress alleviation, supporting amendment-based strategies as a complementary approach to direct inoculation.

Microbial effects can also extend beyond the direct action of individual metabolites through induced systemic tolerance (IST), in which colonization primes the plant for stronger physiological and molecular responses to subsequent stress. Genera such as *Rhizobium*, *Bacillus*, *Pseudomonas*, *Azotobacter*, *Enterobacter*, *Streptomyces*, and *Sphingomonas* contribute to salt tolerance through different and potentially complementary mechanisms (Vaishnav et al., 2019; Kumar et al., 2020; Vaishnav et al., 2016; Gopalakrishnan et al., 2015; Sunita et al., 2020). This supports a microbiome-level view in which the combined functional capacity of microbial communities, rather than a single taxon, underpins resilience to salinity. A caveat is that these studies are dominated by short-term experiments with a single inoculant, and it remains unclear whether the same strains retain activity in soils where salinity co-occurs with drought and nutrient imbalance.

### 2.3 Temperature stress and microbes

Extreme cold and heat destabilize photosynthesis, plasma membranes, enzyme function, and plant growth. Membrane fluidity increases under heat and decreases under cold, and heat stress alters the concentration of the hormone JA. Plants defend themselves through osmolyte accumulation and ROS-scavenging enzymes such as ascorbate peroxidase and catalase, together with heat shock proteins (HSP20, HSP60, HSP70, HSP90, HSP100), but these defenses frequently fail under extreme temperatures, creating a need for additional tolerance mechanisms.

Microbes can support plants at both ends of the temperature range. PGPR colonize the rhizosphere and regulate phytohormones, nutrient acquisition, osmotic balance, antioxidant defense, and stress-responsive gene expression, thereby maintaining photosynthetic activity, improving nutrient uptake, and reducing oxidative damage through ROS scavenging under heat and cold (Zhang et al., 2023). For cold, many micro-organisms aid plant survival at low temperature (Schlaeppi et al., 2014): PGPR isolated from root nodules of pea plants grown in the cold performed as effective biofertilizers at low temperature (Kumar Meena et al., 2015), and *Arthrobacter nicotianae*, *Pseudomonas cedrina*, and *Brevundimonas terrae* maintained plant health in the cold (Yadav et al., 2015). Extending this concept, microbial communities enriched from stress-resistant plants such as pea have been applied to rice, where communities containing nitrogen-fixing bacteria enhanced both cold and heat tolerance while improving soil quality and plant growth (Sarkar et al., 2018).

For heat, *Azospirillum brasilense* and *Bacillus amyloliquefaciens* reduced the pre-activation of heat shock transcription factors, ROS regeneration, and metabolome changes in wheat (*Triticum aestivum*), conferring heat tolerance (Kapoor et al., 2013). Fungal symbionts offer a more specialized route: the endophyte *Curvularia protuberata* and its mycovirus, *Curvularia* thermal tolerance virus (CThTV), enable the panic grass *Dichanthelium lanuginosum* to survive soil temperatures of 38–65 °C in the geothermal areas of Yellowstone National Park, USA (Marquez et al., 2007), and endophytic fungi have likewise induced heat tolerance in wheat, increasing biomass, grain weight, and germination of second-generation seeds (**Figure 4**) (Redman et al., 2002; Hubbard et al., 2014; Poosapati et al., 2014).

**Figure 4:**
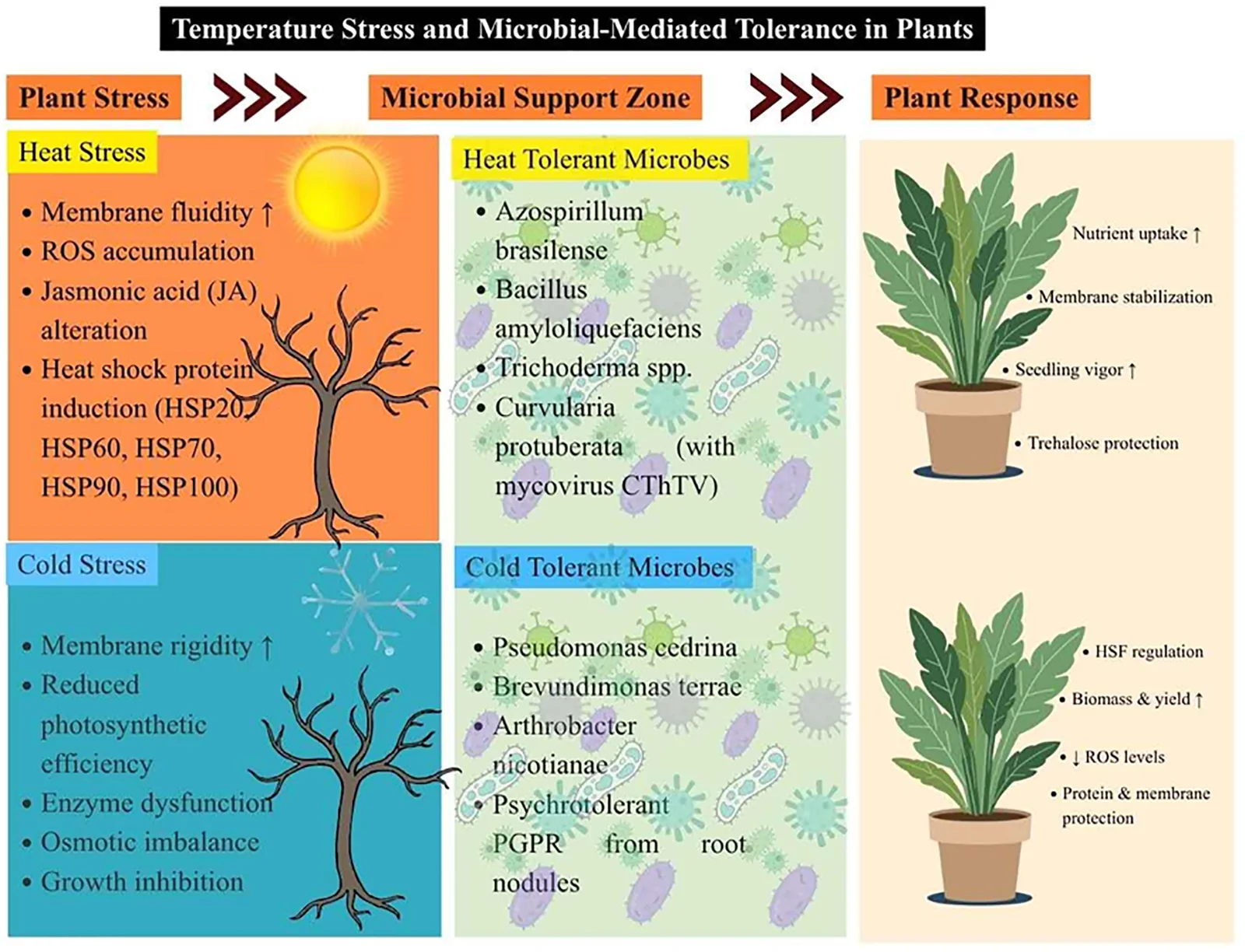
Classification of beneficial microorganisms based on their role in conferring heat and cold stress tolerance in plants. Heat-tolerant microbes regulate oxidative stress, heat shock responses, and metabolic balance, whereas cold-tolerant microbes enhance membrane stability, osmo-protection, and plant growth under low-temperature conditions, ↑ ↓ indicate enhancement and reduction in specific parameters respectively.

Efforts have also been made to select *Trichoderma* isolates from different agro-climatic regions for tolerance to high temperature and salinity, to obtain strains suitable for stress-affected soils (Kapoor et al., 2013). Bacterial heat-stress adaptation is coordinated by the alternative sigma factor σ32 (RpoH), which activates stress-responsive small RNAs such as SorY and StsR in *Rhodobacter sphaeroides* to adjust metabolic flux and cell-cycle progression under thermal and oxidative stress (Yi et al., 2026). This sRNA-based layer of temperature-responsive regulation, documented across diverse bacterial genera including *Escherichia coli*, *Pseudomonas aeruginosa*, and *Rhodobacter*, suggests a possible mechanism by which temperature-tolerant strains selected for microbial inoculant formulations sustain physiological stability during storage and field exposure.

At the cellular level, microbial temperature tolerance is associated with heat shock proteins (HSPs) and cold shock proteins (CSPs), which stabilize proteins, cellular structures, mRNA, and ribosomal activity under temperature extremes (Thieringer et al., 1998). Psychrotrophic and psychrophilic bacteria, including *Pseudomonas putida* and *Sinorhizobium meliloti*, produce CSPs that support mRNA stability and ribosome function at low temperature (Thieringer et al., 1998; Thiagarajan et al., 1992). Compatible solutes add a second layer of protection: trehalose, for example, protects *E. coli* from cold stress by stabilizing cell membranes and preventing protein denaturation, and *Bacillus subtilis* and *Trichoderma* spp. promote the accumulation of trehalose and glycerol, which contribute to membrane stabilization and osmoprotection during heat and cold stress (Maiti et al., 2024). Collectively, phytohormonal regulation, osmo-protection, antioxidant defense, stress proteins, and gene regulation enhance plant resilience to temperature extremes. Two limitations should be noted. Much of the mechanistic evidence on HSPs, CSPs, and trehalose derives from the microbes themselves, so transfer of these benefits to the host is inferred rather than demonstrated, and the *Curvularia*–CThTV system suggests that some tolerance depends on specific, habitat-adapted tripartite associations that simple PGPR inoculation may not reproduce. In addition, compared with drought and salinity, temperature stress has been examined far less with respect to ACC deaminase, EPS, and IST, which represents a clear gap.

### 2.4 Heavy metal tolerance in crop plants

Agricultural soils worldwide are increasingly contaminated through environmental and agronomic pressures, posing a serious threat to food security (Anas et al., 2025). Major sources include urbanization, mining, sewage sludge, and intensive use of agrochemicals and fertilizers (Raklami et al., 2022), and mining in particular releases large volumes of metals into nearby soil (Tepanosyan et al., 2018). Arsenic (As), cadmium (Cd), nickel (Ni), lead (Pb), and zinc (Zn) accumulate in food, water, and air (Rashid et al., 2023), reduce microbial activity, and disrupt nutrient cycling and disease suppression. They also interfere with metabolic and cellular structures in plants, causing stunted growth and oxidative stress, especially in cereals (Vasilachi et al., 2023). The resulting yield loss and compromised food quality extend to humans and animals through the food chain (Javaid et al., 2022), affecting the respiratory tract and cells such as endothelial and epithelial cells (Kiran et al., 2022). Conventional remediation, such as soil excavation and chemical mobilization, is costly, labor-intensive, and may cause secondary pollution, which has directed attention to microbial approaches that reduce both metal bioavailability in soil and metal accumulation in plant tissues (Tiwari and Lata, 2018). Genetically engineered microbes combined with nanotechnology are a recent avenue for improving bioremediation efficiency.

Microorganisms have evolved to survive in contaminated soils and can mitigate metal toxicity and improve growth, with AMF, PGPR, cyanobacteria, and endophytes being the most widely used (as showed in **Table 1**). PGPR such as *Pseudomonas*, *Enterobacter*, *Bacillus*, and *Rhodococcus* are abundant in contaminated soils (Gupta et al., 2024); they fix nitrogen and detoxify metals through bioaccumulation, biosorption, and chelation, and many strains are highly metal-resistant and enhance phytoremediation efficiency. Endophytic bacteria, mainly *Sphingomonas*, *Methylobacterium*, and *Mesorhizobium*, colonize plant tissues, promote growth, and aid detoxification through siderophores and phytohormones that alter metal accumulation. AMF such as *Glomus* and *Rhizophagus*, which associate with about 80% of plants (Ouhaddou et al., 2025), improve nutrient uptake, metal tolerance, and antioxidant defense; their diversity varies with toxicity level and plant species, and fungal-assisted phytoremediation has reduced soil metal contamination (Khalid et al., 2021). Cyanobacteria (*Nostoc*, *Leptolyngbya*) and actinomycetes (*Streptomyces*) are key biocrust components essential for arid ecosystem recovery (Zhu et al., 2025); cyanobacteria-dominated biocrusts immobilize metals through extracellular polymeric substances and improve soil structure and fertility (Pagli et al., 2024), and have reduced arsenic levels by regulating soil microbial activity and physicochemical properties (Mao et al., 2023).

**Table 1:** Microbial bio-stimulants and their mechanistic roles in heavy metal tolerance ↑ ↓ indicate enhancement and reduction in specific parameters respectively.

Crop species	Abiotic stress	Microorganism(s)	Microbial mechanism	Plant response/effect	Reference
A. Multi-metal stress					
Rapeseed (*Brassica napus*)	Heavy metals (Zn, Cu, Cd, Pb)	*Pseudomonas fluorescens* and *Variovorax* sp.	Enhanced phytoextraction	↑ Phytoextraction of Zn, Cu, Cd and Pb	(Dąbrowska et al., 2017)
Soybean (*Glycine max*)	Cr, As	PGPR strain C18, *Bacillus subtilis* and *Trichoderma harzianum*	Phytohormone production (IAA, GA); antioxidant defense; modulation of Cd bioavailability	↑ Antioxidant activity; ↑ IAA and GA; Cd bioavailability; growth enhancement; increased photosynthesis	(Hussain et al., 2021; Haider et al., 2021)
Soybean (*Glycine max*)	Ni, Cd and Al	Endophytic fungi LHL10 and LHL06	Phytohormone regulation (JA, ABA); antioxidant defense; metal-transporter gene regulation	Inhibited metal translocation; downregulation of *gmhma13*, *gmhma14* and *gmhma18*; ↑ antioxidant activity; ↑ ABA	(Bilal et al., 2020)
Soybean (*Glycine max*)	Cd, Co	Cyanobacterium *Synechocystis* PCC6803	Restricted metal translocation to aerial parts	Confined metalloid translocation and accumulation in aerial portions; improved growth; ↑ productivity; ↑ plant survival; ↑ buildup of nutrients	(Armendariz et al., 2019)
Egyptian pea (*Sesbania sesban*)	Cd, Ni, Cr	*Bacillus anthracis* PM21	Growth promotion (mechanism not specified)	Improved seed germination; ↑ root and shoot length; ↑ chlorophyll content	(Ali et al., 2021)
B. Single-metal stress					
Mustard (*Brassica juncea*)	Chromium stress	PGPR strains A3 and S32	Growth promotion (mechanism not specified)	Enhanced growth	(Kumar et al., 2021)
Mustard (*Brassica juncea*)	Ni	*Bacillus subtilis* SJ-101	Growth promotion (mechanism not specified)	Stimulated growth	(Zaidi et al., 2006)
Canola (*Brassica napus*)	Nickel toxicity	*Kluyvera ascorbata* SUD165	Stress protection (mechanism not specified)	Protected canola seedlings	(Chakraborti et al., 2022)
Pepper (*Capsicum annuum*)	Cu	AM fungi	Mycorrhizal symbiosis (mechanism not specified)	Enhanced total dry weight and leaf area	(Ruscitti et al., 2017)
C. Combined metal and salinity stress					
Rice (*Oryza sativa*)	Salt and metal stress	*Pseudomonas stutzeri* A1501	Growth promotion (mechanism not specified)	Enhanced rice growth	(Han et al., 2015)
Maize (*Zea mays*)	Heavy metals and saline-sodic stress	*Ex. aurantiacum*, *P. putida*, *B. sphaericus* and *B. pumilus*	Soil improvement; reduced heavy-metal uptake	Enhanced soil physico-chemical properties; minimized uptake of Cr, Ni and Pb; improved yield	(Ullah and Bano, 2019)

Mechanistically, beneficial rhizospheric microbes improve metal tolerance by regulating phytohormone homeostasis, secondary metabolism, antioxidant defense, gene expression, and metal availability in the rhizosphere. Inoculation modulates stress-related phytohormones and MAPK signaling, a conserved pathway governing abiotic stress perception and downstream defense responses, helping maintain cellular homeostasis under metal exposure (Ali et al., 2022). Directly, microbes improve nutrient acquisition and supply metabolites, vitamins, and minerals while regulating phytohormone levels (Liu et al., 2023; Zhou Y. et al., 2023); ACC deaminase lowers ACC levels, limiting excess ethylene and supporting root and shoot growth, and microbial organic acids modify metal availability and uptake (Prabhakaran et al., 2016; Tiwari and Lata, 2018). Indirectly, microbes induce systemic resistance (ISR) through defense enzymes such as proteases and glucanases (Gul et al., 2023; Nagrale et al., 2023) and activate antioxidant defenses, promoting metal-binding thiols and metallothioneins that support chelation, redox homeostasis, and reduced oxidative damage. Exopolysaccharides, siderophores, organic acids, and amino acids further immobilize metals, alter their mobility, and facilitate nutrient acquisition, thereby balancing immobilization, uptake, translocation, and sequestration (Zhou Y. et al., 2023).

These functions can pull in opposite directions: immobilization lowers metal uptake and toxicity, whereas mobilization by siderophores and organic acids can increase uptake and thus phytoremediation efficiency. The net outcome therefore depends on the metal, soil properties, and the purpose of the system (protecting a food crop versus extracting metals), which may explain why the same trait can be beneficial in one context and counterproductive in another. Collectively, phytohormone regulation, ACC deaminase activity, antioxidant and chelation systems, rhizospheric metal modulation, ISR, and stress-responsive signaling enhance heavy metal tolerance.

### 2.5 Microbial bioremediation and environmental sustainability

Beneficial microorganisms offer promising strategies for bioremediation and restoration of contaminated environments, in addition to alleviating heavy-metal-induced phytotoxicity. Through biosorption, bioaccumulation, biotransformation, precipitation, and intracellular or extracellular sequestration, microbes reduce metal toxicity and mobility. These processes can be coupled with phytoremediation, whereby metal-resistant rhizobacteria modify rhizosphere metal availability while promoting plant growth and facilitating phytoextraction or metal immobilization.

Advances in microbial engineering have further expanded the potential for targeted metal sequestration. For example, Gahlot et al. (2020) engineered *E. coli* to express Cu²^+^-binding peptides, enhancing copper tolerance and metal-binding capacity. Although environmental deployment of such engineered systems requires careful ecological and biosafety evaluation, they illustrate the promise of tailored biological platforms for metal recovery and remediation.

Microbial remediation must also be integrated with agricultural waste and wastewater management to ensure environmental sustainability. Agricultural residues can serve as microbial carriers or soil amendments, enabling waste valorization alongside the restoration of degraded soils. Conversely, improper discharge of agricultural effluents continually introduces heavy metals, impairing soil health and destabilizing agroecosystems, making effluent management essential for source control. Kukreja et al. (2012) highlighted the environmental impacts of distillery spent wash and examined biological treatments to reduce its pollutant load. Properly treated effluents may supply organic matter and nutrients for agricultural use; however, application rates must be carefully regulated, as excessive loading can adversely affect soil properties, salinity, and plant growth. Combining microbial remediation, phytoremediation, waste valorization, and appropriate effluent treatment can therefore support soil restoration, resource recovery, and sustainable agroecosystem management. Future research should evaluate these approaches under field conditions, with emphasis on long-term pollutant immobilization or removal, soil health, microbial stability, crop safety, and environmental sustainability.

## 3 Legume-rhizobium symbiosis and inoculation strategies under abiotic stress

The legume-rhizobium symbiosis underpins global food production and nitrogen cycling. It is thought to have originated from a common ancestor within the nitrogen-fixing clade (Fabales, Fagales, Cucurbitales, Rosales) approximately 92–110 million years ago (Soltis et al., 1995; Wang et al., 2009; Bell et al., 2010). Although this ancestral lineage lacked nodulation, subsequent evolutionary innovations enabled nodule formation and conserved infection mechanisms such as cortical infection threads (Cathebras et al., 2022), while nodule structure, infection strategy, and host-symbiont specificity have since diversified considerably (Masson-Boivin and Sachs, 2018).

Nodulation begins with a molecular dialogue in which host-derived flavonoids induce rhizobial Nod factor synthesis, triggering root-hair curling, infection-thread formation, and nodule organogenesis (Suzaki and Kawaguchi, 2014). Within nodules, rhizobia differentiate into bacteroids and fix atmospheric N_2_ in exchange for host-derived carbon, directly supporting plant nitrogen nutrition and productivity.

Symbiotic efficiency, however, depends strongly on rhizobial competitiveness and environmental conditions. Native rhizobia often outcompete introduced inoculants for nodule occupancy (Onishchuk et al., 2017), and abiotic stresses such as drought, salinity, heat, and acidity impair rhizobial survival, colonization, and nitrogenase activity (Atieno and Lesueur, 2019). Effective inoculants must therefore combine high fixation efficiency with host compatibility, competitiveness, and stress tolerance. Consistent with this, Shah et al. (2024) generated γ-irradiation-derived mutants (MbPrRz1-3; identified as *Mesorhizobium ciceri* and *Rhizobium leguminosarum*) that outperformed indigenous and commercial strains in chickpea, field pea, and lentil, improving nodulation, yield, and soil nutrient status while expressing the *nifH* marker. Complementary management practices can further enhance inoculant performance. Co-inoculation with PGPR or mycorrhizal fungi can couple N_2_ fixation with improved nutrient acquisition and stress resilience, while soil amendments can modulate rhizosphere conditions to favor nodulation. For instance, Gahlot et al. (2011) reported that digested distillery spent wash influenced chickpea nodulation and nutrient uptake in a concentration- and application-dependent manner, with soil application outperforming irrigation. Collectively, these findings highlight the value of integrating stress-tolerant, competitive rhizobial strains with compatible microbial consortia and optimized delivery systems to strengthen biological nitrogen fixation and reduce reliance on synthetic fertilizers.

## 4 The enrichment of beneficial microorganisms to enhance crops’ tolerance to abiotic stress

Agriculture is engaging more than 50% of the population’s economic activity. The frequent use of fertilizers and pesticides causes low productivity, food poisoning, and soil damage. Microbial inoculants have the capacity to enhance nutrient uptake and promote plant sustainable yield (Wei et al., 2024; Leggett et al., 2011). Agronomic practice also contributes to improving soil health and induces systemic resistance by introducing beneficial microorganisms in the rhizosphere and warding off pathogenic invasions (Ribeiro et al., 2022). In the rhizosphere, inoculants such as *rhizobium*, *Bacillus spp*, and *Azospirillum* have effectively improved nutrient uptake, root dry weight, and increased tolerance to environmental constraints (de Sousa et al., 2021; Ribeiro et al., 2022). The inoculation at sowing boosts yields and also reshapes the soil microbial community, such as 28% yield improvement using such a method in soybean (Moretti et al., 2018). The direct nitrogen fertilizer application and organic amendments enhance microbial population by reducing the negative effects of excessive chemical fertilizer use and improving overall soil health compared to the water regime (Li et al., 2023).

Effective microorganism is sprayed in a foliar manner on the leaf surface to get enriched, typically in the morning or late afternoon, to reduce injury chances. These microbial consortia help to reduce harmful leaf pathogens and enhance beneficial phyllo sphere microbial pollutions. These inoculants enhance yield by stress alleviation and nutrient improvement, with bacteria like *Pseudomonas* and *Bacillus* having high effectiveness. These interventions improve nutrient uptake, enhancing crop quality and supporting sustainable yield across cropping systems as illustrated in (**Figure 5**).

**Figure 5:**
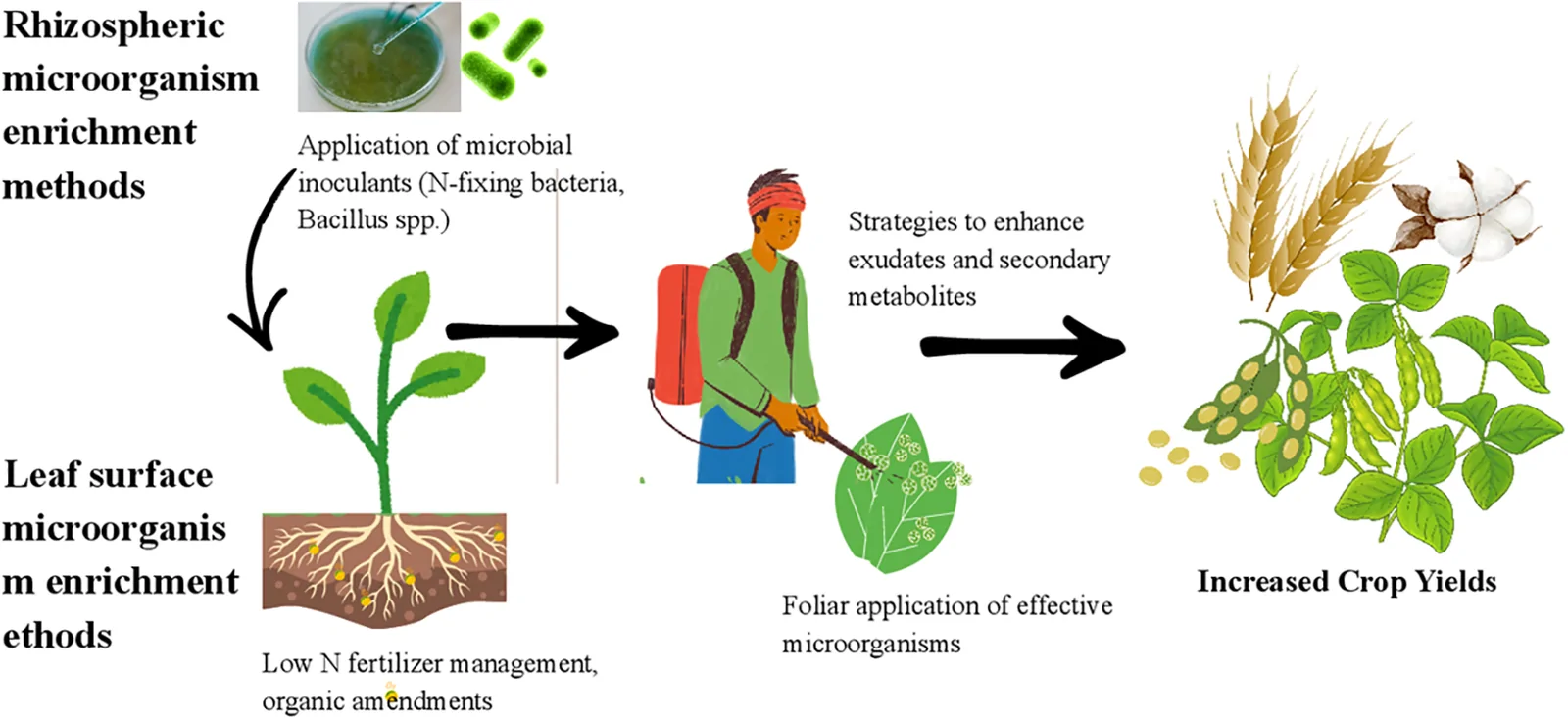
Schematic representation of agronomic microbial enrichment methods, foliar application of beneficial microorganisms, and nutrient management practices that enhance plant microbe interactions and increase crop productivity.

Microbial inoculants often promote plant growth and stress tolerance under controlled and greenhouse conditions, but their efficacy and stability generally decline once they are deployed in the field. In a meta-analysis of 71 studies, the largest effects were recorded on sterilized substrates (lnRR = 0.29). Effects were smaller in unsterilized substrates and field experiments (lnRR = 0.23 and 0.22, respectively, and they declined with experimental duration from 0.33 in experiments shorter than one month to 0.12 in those longer than three months (Azarbad and Junker, 2024). A major reason is competition with native microorganisms. Sterilization removes competitors and so magnifies inoculation effects, whereas in non-sterile soil the inoculant must colonize.

The rhizosphere and persist among indigenous communities (Azarbad and Junker, 2024; Romano et al., 2020). Persistence is frequently limited. *Trichoderma harzianum* and *Bacillus amyloliquefaciens* FZB42 fell below detectable limits within a few weeks of application and rhizobia may persist indefinitely but at abundance lower than those required for that efficacy (Parnell et al., 2016). Fluctuating soil properties further modulate performance. Effect sizes correlated negatively with soil pH (R = −0.21), total nitrogen (R = −0.17) and total phosphate (R = −0.57), and positively with organic matter (R = 0.42) and electrical conductivity (R = 0.45) (Azarbad and Junker, 2024). Climatic variables also matter as, temperature and light significantly affected biocontrol, by nonpathogenic *Fusarium oxysporum* (Larkin and Fravel, 2002, cited in Parnell et al., 2016). Additionally, products stored under high temperature, light and humidity require robust formulation to maintain shelf life (Parnell et al., 2016). Field responses are consequently positive but variable. *Penicillium bilaii* raised maize yield significantly in 72% of small plots and 80% of large plots, yet mean gains were only 1.8% and 3.5% (Leggett et al., 2015, cited in Parnell et al., 2016). *Azospirillum* inoculation increased yields in 60–70% of trials (Okon and Labandera-Gonzales, 1994). Distinguishing inoculants from native populations in non-sterile soil remains technically difficult, and tagged strains are restricted to laboratory or greenhouse work (Romano et al., 2020). Field validation across sites and seasons, with persistence monitored, is therefore essential.

## 5 Rhizosphere engineering: an underdeveloped frontier

Rhizosphere engineering, the deliberate manipulation of plant, microbial, and soil components of the root zone to improve productivity and stress resilience, remains only partially integrated into the biofertilizer literature (Ahkami et al., 2017; Hakim et al., 2021).

### 5.1 Host-mediated microbiome recruitment

Plants shape and are shaped by their rhizosphere communities through a bidirectional feedback loop in which rhizodeposition alters microbial composition, which in turn influences plant growth and productivity (Dessaux et al., 2016; Philippot et al., 2013, as reviewed in Hakim et al., 2021). Community structure is determined jointly by plant species identity and soil plantation history, so host genotype and cropping history leave an exploitable microbiome legacy (Schmid et al., 2018). Mechanistically, systemically induced root exudation of metabolites (SIREM) links colonization at one root location to altered exudation elsewhere (Korenblum et al., 2020; Wankhade et al., 2025), and jasmonic-acid signaling similarly reprograms exudate composition and the associated bacterial/archaeal community (Carvalhais et al., 2015). Interplant signaling can also recruit protective taxa: in a tomato–potato–onion intercrop, root-exuded taxifolin recruited *Bacillus* spp. to the tomato rhizosphere and suppressed *Verticillium dahliae* (Zhou, X. et al., 2023).

### 5.2 Root exudate engineering

Transgenic rice and tomato overexpressing the *Arabidopsis* vacuolar H^+^ pyrophosphatase gene *AVP1* released ~50% more citrate and malate under AlPO_4_ treatment, enhancing Al³^+^ tolerance and phosphorus acquisition (Yang et al., 2007; Ahkami et al., 2017). More recently, altering transporter expression to modify malate, citrate, and γ-aminobutyric acid release from root apices produced spatially resolved shifts in root-associated microbiome composition (Kawasaki et al., 2021; Wankhade et al., 2025). Exudates also act as chemoattractants: nucleosides attract beneficial *Bacillus*/*Pseudomonas* but also some pathogens (Keren et al., 2024), arginine shapes community assembly in *Casuarina equisetifolia* (Lin et al., 2022), and the maize benzoxazinoid DIMBOA selectively recruits *Pseudomonas putida* KT2440 (Neal et al., 2012). Transporter modification or breeding for altered exudate chemistry is therefore a tractable but underexploited lever.

### 5.3 Synthetic microbial communities and designer microbiomes

Community design is constrained by ecological complexity: even a two-strain co-culture involves six possible interaction types (Grosskopf and Soyer, 2014), and a three-strain consortium already yields >729 predicted interactions. Ahkami et al. (2017) therefore propose engineering a genetically tractable *Bacillus* chassis with nitrogen-fixation genes (e.g., *nifH* from *Paenibacillus*) or pairing it with natural diazotrophs (*Pseudomonas*, *Rhizobium*, *Bradyrhizobium*), using engineered metabolic interdependency (e.g., cross-feeding of essential co-factors) to favor cooperation over competition. Candidate strains are selected for colonization efficiency, competitiveness, root attachment, chemotactic responsiveness, and the ability to reach threshold densities (e.g., 10^5^–10^6^ CFU g^-^¹ root for *Pseudomonas*-mediated biocontrol; Haas and Défago, 2005, cited in Ahkami et al., 2017).

Wankhade et al., (2025) extend this to designer microbiomes assembled from genomically and phenotypically screened strains (e.g., *Bacillus*, *Pseudomonas*, *Azospirillum*) selected for osmoprotectant, exopolysaccharide, or ACC-deaminase production. Reported outcomes are substantial: a *Haloxylon ammodendron* rhizosphere-derived SynCom improved maize water-use efficiency by up to 700% under drought in greenhouse and field trials (Nourashrafeddin et al., 2024); a four-species *Arabidopsis* SynCom (*Stenotrophomonas*, *Xanthomonas*, *Microbacterium*, *Paenibacillus*) conferred drought tolerance through emergent community-level properties such as synergistic biofilm formation (Yang, N. et al., 2021); and a cotton SynCom applied as a seed dressing increased germination by 14.3% and yield by 8.5%, while soil application increased nitrate availability by up to 55% (Kaur et al., 2022). In soybean, SynCom inoculation systemically regulated nitrogen- and phosphorus-signaling networks and upregulated auxin-responsive genes, indicating that designer consortia act through host signaling reprogramming as well as nutrient supply (Wankhade et al., 2025).

### 5.4 Targeted microbiome manipulation and precision agriculture

In contrast to broad-spectrum biofertilizers aimed at general soil fertility, “microbial vaccines” introduce a specific beneficial taxon (e.g., the diazotroph *Azotobacter*) to elicit a defined outcome such as induced immunity or nitrogen supply (Wankhade et al., 2025). Field translation remains a major bottleneck, however: native soil microbiota frequently out-compete introduced strains, and community dynamics observed *in vitro* often fail to reproduce under field heterogeneity in soil type, climate, and management (Wankhade et al., 2025). Overcoming this requires identifying stable core taxa that persist across environments (Rodrigues et al., 2018) and integrating rhizosphere engineering with precision agriculture tools, including remote sensing, machine learning-based soil microbiome diagnostics, and site-specific inoculant recommendations matched to local edaphic and climatic conditions (Wankhade et al., 2025). Soil amendments (biochar, silicon, zeolites, manure) remain a complementary but largely empirical lever for inoculant establishment (Hakim et al., 2021), underscoring that microbiome, exudate, and soil engineering must be integrated rather than pursued in isolation.

## 6 Modern omics and systems biology approaches in plant–microbe interactions

Integrated, high-throughput omics platforms now resolve plant–microbe interactions at genomic, transcriptomic, proteomic, and metabolomic scales, providing mechanistic rather than purely descriptive insight into microbe-mediated stress tolerance and plant growth promotion (Jain et al., 2024; Kumar et al., 2023).

### 6.1 Genomics and metagenomics

Next- and third-generation sequencing (Illumina, Oxford Nanopore, PacBio) has made whole-genome and shotgun metagenomic sequencing routine for characterizing inoculant strains and their rhizosphere/endosphere communities, including unculturable members, and for resolving functional genes underlying plant growth promotion, such as *nif* operons, *acdS* (ACC deaminase), and phosphate solubilization loci (Jain et al., 2024; Enespa and Chandra, 2022). Functional-gene arrays (GeoChip) and 16S/ITS amplicon profiling further enable culture-independent assessment of inoculant establishment and persistence in soil (Enespa and Chandra, 2022).

### 6.2 Transcriptomics and proteomics

RNA-seq and dual RNA-seq permit simultaneous profiling of host and microbial transcriptomes during colonization, capturing reciprocal gene-expression dynamics and the role of non-coding RNAs in modulating induced systemic resistance and stress signaling (Imam et al., 2016; Jain et al., 2024). LC-MS/MS-based meta-proteomics complements these data by directly evidencing protein expression, post-translational modification, and protein–protein interaction networks during inoculant–root interactions (Enespa and Chandra, 2022; Jain et al., 2024).

### 6.3 Metabolomics

NMR- and MS-based (GC-MS, LC-MS) metabolomics is particularly powerful for elucidating the biochemical basis of symbiosis, capturing exudate-mediated recruitment, strain-specific secondary metabolite responses, and rhizosphere signaling molecules (e.g., flavonoids, organic acids, quorum-sensing compounds) that govern inoculant compatibility and efficacy under abiotic stress; MALDI mass spectrometry imaging additionally localizes these metabolites within colonized root and nodule tissue (Gupta et al., 2022).

### 6.4 Multi-omics integration and systems biology

Because no single omics layer fully explains inoculant performance, integration across omics layers, coupled with genome-scale metabolic modeling of plant and microbial partners, is increasingly used to reconstruct metabolic cross-talk during beneficial associations and to predict consortium-level outcomes *in silico* (Imam et al., 2016; Kumar et al., 2023). Such integration is central to translating mechanistic understanding into sustainable agricultural applications, including rational design of microbial consortia and biofertilizer formulations (Kumar et al., 2023).

### 6.5 Emerging directions

Complementary advances in super-resolution and volume electron microscopy, X-ray tomography, and correlative light electron microscopy now allow nanoscale, spatially resolved visualization of inoculant colonization structures (e.g., arbuscules, biofilms) that anchor omics data to cellular architecture (Czymmek et al., 2023), although applications of volume EM specifically to plant-microbe interaction sites remain scarce. Looking forward, single-cell and spatial multi-omics, together with machine learning-based integration of these large, heterogeneous datasets, are expected to resolve strain level and cell type specific heterogeneity in inoculant host interactions and to accelerate predictive modeling of formulation performance across environments. For example, single-nucleus and spatial transcriptomics of *Medicago truncatula* roots colonized by *rhizophagus irregularis* resolved distinct transcriptional states in cortex cells across successive stages of colonization (Serrano et al., 2024), and single-cell profiling of M. *truncatula* nodules resolved 13 cell clusters, showing that uninfected cells in the nitrogen-fixation zone contribute to nitrogen assimilation through asparagine synthesis (Ye et al., 2022).

Together, these converging omics and systems level approaches move microbial inoculant research from empirical formulation toward mechanistically guided, predictive design of biofertilizer products.

## 7 Stress-tolerant microbes in Industrial and agricultural applications

Biofertilizers are formulated products of living microorganisms capable of converting nutrients from unusable to usable forms through biological processes. Under abiotic and biotic stress, biofertilizers are used to enhance plant growth (**Table 2**). PGPR can convert atmospheric nitrogen into ammonia, mobilize nutrients within the plant rhizosphere, and solubilize phosphate (Kantachote et al., 2016). Common genera such as *Azotobacter*, *Acetobacter*, *Azospirillum*, *Pseudomonas*, and *Bacillus* are well-characterized plant growth-promoting strains that also function as potent biocontrol agents under stress conditions (Kumar et al., 2014). Biofertilizers have been used globally for decades, and multifunctional formulations now represent an efficient substitute for synthetic pesticides and inorganic fertilizers, offering a route to improved soil health. Continued selection of stress-tolerant microorganisms and refinement of biofertilizer formulation strategies will likely prove advantageous for environmental reclamation going forward. The diversity of beneficial soil microbes may facilitate ecosystem sustainability owing to their cosmopolitan distribution, stress-tolerance potential, broad genetic pool, and catabolic versatility.

**Table 2:** Beneficial microorganisms and their mechanisms in enhancing abiotic stress tolerance in crop species.

Crop species	Stress condition	Microorganism(s)	Microbial mechanism	Plant response/effect	Reference
A. Salinity stress					
Wheat (*Triticum aestivum*)	Salt stress	*Acinetobacter*, *Bacillus*, *Enterobacter*, *Pantoea*, *Sinorhizobium*, *Marinobacterium*, *Pseudomonas*, *Rhizobium*	Growth promotion (mechanism not specified)	Enhanced seed germination and vigor index	(Sorty et al., 2016)
Rice (*Oryza sativa*)	Saline stress	Root-associated PGPRs	Regulation of stress-responsive gene expression	Expression of the *RAB18* gene linked to salt stress	(Jha et al., 2014)
Chickpea (*Cicer arietinum*)	Salt stress	*Bacillus subtilis* BERA 7	Stress alleviation (mechanism not specified)	Reduced salt stress effect	(Abd-Allah et al., 2018)
Maize (*Zea mays*)	Salt stress	*P. geniculate* MF-84	Ion homeostasis in roots: reduced Na^+^ uptake; increased K^+^ and Ca²^+^ uptake	Maintained ionic balance/homeostasis; ↑ plant growth; decreased crop losses	(Singh et al., 2020)
Wheat (*Triticum aestivum*)	Salt stress	*P. polymyxa*, *B. pacificus* and *B. nakamurai*	Antioxidant enzyme activity; osmolyte (proline) modulation	Increased relative water content, electrolyte leakage and enzymatic activity; reduced proline accumulation in shoots; improved antioxidant enzymes, growth and yield	(Saad et al., 2019)
Lettuce seeds (*Lactuca sativa*)	NaCl exposure	*Azospirillum*	Growth promotion (mechanism not specified)	Enhanced seed germination; ↑ vegetative growth	(Samaddar et al., 2019)
Lettuce (*Lactuca sativa*)	Salt stress	*Pseudomonas mendocina*, alone or co-inoculated with *Glomus mosseae*/*Glomus intraradices*	Improved nutrient uptake; PGPR–AMF co-inoculation	↑ growth; ↑ nutrient uptake; ↑ other physiological activities	(Kerbab et al., 2021)
B. Temperature stress					
Tomato seedlings (*Solanum lycopersicum*), groundnut (*Arachis hypogaea*) and pepper (*Capsicum annuum*)	Temperature (not specified)	*Achromobacter piechaudii* ARV8, *Bacillus* sp. and *Pseudomonas fluorescens*	Growth promotion (mechanism not specified)	Improved seedling growth	(Juric et al., 2020)
Canola (*Brassica napus*)	Low temperature: 5 °C treatment during both spring and winter	*Pseudomonas putida* GR12-2	Growth promotion; root development	Promoted growth and root enlargement	(Yang et al., 2020)
Soybean (*Glycine max*)	Suboptimal root-zone temperatures	*Serratia liquefaciens*, *Serratia proteamaculans* and *Bradyrhizobium japonicum*	Growth and physiological promotion (mechanism not specified)	Beneficial effects on growth and physiology	(Yang et al., 2020)
Grapevine (*Vitis vinifera*)	Cold stress	*Burkholderia phytofirmans* PsJN	Membrane integrity maintenance	Lowered biomass reduction and membrane electrolyte leakage	(Yang et al., 2018)
Tomato (*Solanum lycopersicum*)	Chilling stress	AM *Glomus mosseae*	Antioxidant defense; osmotic adjustment; photosynthetic pigment maintenance	Reduced membrane lipid peroxidation; enhanced photosynthetic pigment levels; accumulation of osmotic adjustment compounds; increased antioxidant enzyme activity	(Sytar et al., 2019)
Soybean (*Glycine max*)	Heat stress	*Bacillus tequilensis* SSB07	Growth promotion (mechanism not specified)	Increased shoot length and biomass	(Kang et al., 2019)
Wheat (*Triticum aestivum*)	High-temperature stress	Strain SN13 (microbial inoculation)	Regulation of phytohormones and stress-responsive genes	Positively regulated phytohormone and stress-gene expression, indicating a multifaceted stress response; improved membrane potential, osmolyte buildup, proline content, lipid peroxidation, total soluble sugar and other physiological and biochemical variables	(Maitra et al., 2021)
Tomato (*Solanum lycopersicum*)	Heat stress	*Paraburkholderia phytofirmans* strain PsJN	Photosynthetic performance; metabolite accumulation	Improved plant growth attributes: chlorophyll content, photosystem II efficiency, and sugar and total protein content	(Issa et al., 2018)
C. Drought and water deficit					
Maize (*Zea mays*)	Severe drought for 15 days	*Burkholderia phytofirmans* and *Enterobacter* sp. FD1	Enhanced photosynthesis	Enhanced photosynthesis, shoot biomass and root biomass	(Naveed et al., 2014)
Rice (*Oryza sativa*)	Water deficiency	*Gluconacetobacter diazotrophicus* Pal5	Antioxidant and osmolyte-related gene expression	Expression of *gor*, *cat*, *P5CR*, *sod* and *BADH*; ↑ proline; ↑ glycine betaine	(Filgueiras et al., 2020)
Maize (*Zea mays*) and sorghum (*Sorghum bicolor*)	Drought	*Azospirillum brasilense* and *Bacillus subtilis*	Improved water use efficiency; root growth stimulation	Yield improved by 20%; water use efficiency enhanced by 15%; increased root growth	(Galindo et al., 2022)
Cereals	Drought	*Azotobacter* sp., *Pseudomonas putida* and Acetobacteraceae	Nutrient availability: nitrogen fixation and phosphate solubilization	Improved nutrient availability	(Kálmán et al., 2024; Swift et al., 2024)
Soybean (*Glycine max*) and potato (*Solanum tuberosum*)	Drought	*Bacillus polymyxa*, *Glomus intraradices* and *Glomus versiforme*	Osmolyte accumulation	Increased production of osmolytes (proline and phenolics)	(Vurukonda et al., 2016)
D. Heavy-metal and combined stress					
Rice (*Oryza sativa*)	Salt and metal stress	*Pseudomonas stutzeri* A1501	Growth promotion (mechanism not specified)	Enhanced rice growth	(Han et al., 2015)
Maize (*Zea mays*)	Heavy metals and saline-sodic stress	*Ex. aurantiacum*, *P. putida*, *B. sphaericus* and *B. pumilus*	Soil improvement; reduced heavy-metal uptake	Enhanced soil physico-chemical properties; minimized uptake of Cr, Ni and Pb; improved yield	(Ullah and Bano, 2019)

Microbial approaches to stress mitigation are increasingly considered alongside other emerging technologies, including nanoparticles and nano-enabled formulations (Abdulraheem et al., 2025). Nanoscale materials metal oxide, biopolymer-based, and nanocomposite carriers among them have been explored for controlled nutrient release, targeted delivery of agrochemicals, and protection of bioactive compounds against environmental degradation, properties that parallel several of the functions sought in microbial inoculant design. While the present review focuses on microbial mechanisms of stress tolerance rather than on nanomaterials themselves, this convergence raises a reasonable question for future formulation science: whether combining microbial inoculants with nanoscale delivery systems could offer complementary benefits, such as improved storage stability, protection from desiccation, or more controlled release of encapsulated cells, under field conditions. This remains a comparatively underexplored area, and any such combination would need to be evaluated against the same criteria applied to conventional carriers cell viability during formulation and storage, compatibility of the nanomaterial with microbial physiology, cost-effectiveness at scale, and regulatory considerations before it could be regarded as a genuine advance over existing encapsulation and immobilization strategies rather than simply an additional layer of complexity.

PGPR have also been identified as microbial agents capable of supporting plant growth while withstanding environmental stress. As non-renewable energy sources gradually become depleted, lignocellulose-based bioethanol production offers a sustainable alternative to conventional fossil fuels, and bioethanol has been extensively investigated as a substitute biofuel; production typically relies on fermentation by thermotolerant or thermophilic microbial strains. Separately, the accumulation of heavy metals in agricultural produce is associated with blood- and skin-related disorders in humans upon consumption. Microorganisms contribute to the remediation of these toxic elements by facilitating their removal from soil and reducing their uptake by plants, offering a further point of convergence with the broader suite of stress-mitigation technologies discussed above.

### 7.1 Commercialization and field application challenges of PGPR

Despite a substantial biofertilizer and bio-stimulant sector (du Jardin, 2015; Backer et al., 2018), translating laboratory efficacy into consistent field performance remains limited by interacting biological, technological, regulatory, and socio-economic constraints (Timmusk et al., 2017; Santos et al., 2019).

### 7.2 Environmental variability and inconsistent field efficacy

PGPR performance is strongly context-dependent. A global meta-analysis reported mean yield gains of +20.0 ± 1.7% in dry, +14.9 ± 1.2% in tropical, +10.0 ± 3.7% in oceanic, and +8.5 ± 2.4% in continental climates, with wide variation among studies (Schütz et al., 2018). Likewise, yield increases from selected *Azospirillum brasilense* and *A. lipoferum* strains in Brazilian maize and wheat were not reproduced at all sites (Hungria et al., 2010), and many commercial phosphorus-solubilizing inoculants gave inconsistent or non-significant effects owing to soil P status, native microbiota, and poor strain persistence (Owen et al., 2015). This variability is increasingly interpreted through invasion ecology: introduced strains must establish against adapted resident communities under temperature, moisture, pH, competition, and predation constraints (Martínez-Viveros et al., 2010; Mawarda et al., 2020), while traits selected for inoculant production, such as rapid growth and high rhizosphere competence, may conflict with those required for persistence and safety (Kaminsky et al., 2019). The greater reliability of rhizobial legume inoculants, whose host specificity and nodule occupancy provide a protected niche (Bashan et al., 2014), suggests that persistence, rather than intrinsic plant-growth-promoting capacity, is the primary determinant of field success.

### 7.3 Host and genotype specificity

Many PGPR are effective only with particular species or cultivars (Dhawi and Hess, 2017; Pratush et al., 2018; Ma et al., 2020), as host genotype shapes root exudation and rhizosphere recruitment, so a strain beneficial to one cultivar may be neutral or inhibitory to another. Identifying optimal strains for each crop–soil combination thus requires costly multi-location, multi-season testing. Synthetic communities of compatible, functionally complementary strains may partly buffer this specificity but add compatibility, regulatory, and formulation challenges (Backer et al., 2018).

### 7.4 Limitations of the screening pipeline

Candidate PGPR are typically selected on *in vitro* traits such as indole-3-acetic acid production, phosphate solubilization, and siderophore production (Ganesh et al., 2022), which poorly predict field performance because expression in culture differs from that in the rhizosphere, while strains lacking these traits may act through poorly characterized mechanisms and be discarded early (Cardinale et al., 2015). Because most evidence, including that for endophytes, derives from greenhouse or growth-chamber studies (Timmusk et al., 2017), pipelines should incorporate early *in planta* assays, rhizosphere competence tests, and multi-environment field validation.

### 7.5 Formulation technologies for commercial inoculants

Formulation determines whether a viable, active inoculum reaches the plant and is the main technological bottleneck in PGPR commercialization (Bashan et al., 2014; Herrmann and Lesueur, 2013). No single type (liquid, wettable powder and other dry formulations, granules, or encapsulated slow-release products) is optimal: liquids are easiest to apply but have the shortest shelf life, whereas carrier-based and encapsulated products improve stability at the expense of production complexity and cost (Bashan et al., 2014; Schoebitz et al., 2013). Selection should therefore consider the organism (spore-formers such as *Bacillus* tolerate drying far better than most Gram-negative genera), crop, application equipment, and target market.

### 7.6 Shelf life and storage stability

As living products, PGPR are limited by viability losses during production, storage, and transport (Arriel-Elias et al., 2018), which depend on taxon, water activity, storage temperature, and protective additives (Berninger et al., 2018). Spore-forming genera are generally more storage-stable than non-spore-forming Gram-negative bacteria, explaining the predominance of *Bacillus*-based products. Protectants, stabilized drying, and encapsulation extend shelf life but raise costs, a particular constraint for smallholders lacking cold storage (Bashan et al., 2014). Because viability does not guarantee activity, physiological function after storage should also be assessed (Berninger et al., 2018).

### 7.7 Regulatory hurdles and lack of standardization

Regulatory frameworks for microbial products differ widely between jurisdictions and often lag behind the science. In the European Union, microbial biostimulants fall under Regulation (EU) 2019/1009, which limits both claims and permitted genera, whereas elsewhere products may be classified as biofertilizers, biopesticides, or plant supplements with different data requirements, increasing registration costs and delaying market entry (du Jardin, 2015; Backer et al., 2018). Inconsistent quality control, including lower-than-claimed cell counts and contamination (Owen et al., 2015), further erodes farmer confidence, underscoring the need for harmonized standards for strain identification, minimum viable counts at end of shelf life, and efficacy testing.

### 7.8 Economic considerations and farmer adoption

Adoption depends on a demonstrable and reliable return relative to conventional inputs. Farmers often perceive PGPR products as costlier and less reliable than chemical fertilizers, a view reinforced by inconsistent field results (Timmusk et al., 2017), and risk aversion arising from limited financial buffers between seasons discourages non-traditional inputs (Parnell et al., 2016). The rapid uptake of rhizobial inoculants in Brazilian soybean production shows that adoption follows when performance is consistent, cost is low relative to benefit (including nitrogen fertilizer replacement), and extension support is available (Hungria et al., 2010; Santos et al., 2019). Farmer training, on-farm demonstration trials, and transparent efficacy data are therefore essential.

## 8 Conclusion

Climate change driven abiotic stresses such as drought, salinity, extreme temperatures, and heavy metal pose an intensifying threat to crop productivity and global food security. Collectively, the evidence reviewed here demonstrates that plant-associated microorganisms, particularly arbuscular mycorrhizal fungi and plant growth-promoting rhizobacteria, function as vital ecosystem components that actively improve plant resilience to these stresses. Their shielding mechanisms are diverse and often synergistic, encompassing the modulation of phytohormone signaling, osmolyte accumulation, extracellular polymeric substance secretion, antioxidant enzyme activity, and the establishment of symbiotic interfaces that improve nutrient and water acquisition. When deployed as multi-strain consortia rather than single isolates, these microorganisms frequently confer broader-spectrum tolerance across simultaneous biotic and abiotic stress conditions, while also minimizing dependence on synthetic pesticides and fertilizers advancing the parallel goals of stress mitigation and soil sustainability.

Despite this promise, significant knowledge gaps constrain the translation of microbial stress-amelioration strategies from controlled settings to reliable field-scale application. The mechanistic basis of many plant-microbe-stress interactions remains incompletely characterized, particularly regarding how microbial consortia coordinate function under fluctuating, multi-stress environments rather than the single-stress conditions typical of laboratory studies. Microbial efficacy is also highly context-dependent, varying with soil type, native microbiome composition, crop genotype, and climatic zone yet comparative data across arid and non-arid regions remain sparse. Furthermore, inconsistencies in inoculant formulation, viability, and field persistence continue to undermine reproducibility between experimental and applied outcomes.

Future research should therefore prioritize (1) multi-omics characterization of plant–microbe signaling networks under combined and sequential abiotic stresses; (2) bioprospecting of underexplored arid and marginal soils to capture stress-adapted microbial diversity; (3) standardized field trials of consortium performance across diverse agroecological zones to bridge the gap between greenhouse and field efficacy; and (4) integration of microbial inoculants with conventional breeding and biotechnological approaches to develop climate-resilient crop management systems. Addressing these priorities will be essential to establishing microbial-based interventions as a scientifically validated, scalable pillar of sustainable agriculture and global food security policy.

The use of microbial inoculants for stress mitigation is part of a broader movement toward regenerative agriculture, in which biological processes are harnessed to restore soil function. Biocrusts, for example, play important roles in soil stabilization and nutrient cycling in dryland systems (Gufwan et al., 2025). Understanding how biocrust communities respond to stress and how they interact with crop plants could inform the design of more resilient agricultural systems.
